# Japanese Medaka: A Non-Mammalian Vertebrate Model for Studying Sex and Age-Related Bone Metabolism *In Vivo*

**DOI:** 10.1371/journal.pone.0088165

**Published:** 2014-02-11

**Authors:** Admane H. Shanthanagouda, Bao-Sheng Guo, Rui R. Ye, Liang Chao, Michael W. L. Chiang, Gopalakrishnan Singaram, Napo K. M. Cheung, Ge Zhang, Doris W. T. Au

**Affiliations:** 1 State Key Laboratory in Marine Pollution, Department of Biology and Chemistry, City University of Hong Kong, Hong Kong; 2 Institute for Advancing Translational Medicine in Bone & Joint Diseases, School of Chinese Medicine, Hong Kong Baptist University, Hong Kong; National University of Singapore, Singapore

## Abstract

**Background:**

In human, a reduction in estrogen has been proposed as one of the key contributing factors for postmenopausal osteoporosis. Rodents are conventional models for studying postmenopausal osteoporosis, but the major limitation is that ovariectomy is needed to mimic the estrogen decline after menopause. Interestingly, in medaka fish (*Oryzias latipes*), we observed a natural drop in plasma estrogen profile in females during aging and abnormal spinal curvature was apparent in old fish, which are similar to postmenopausal women. It is hypothesized that estrogen associated disorders in bone metabolism might be predicted and prevented by estrogen supplement in aging *O. latipes*, which could be corresponding to postmenopausal osteoporosis in women.

**Principal findings:**

In *O. latipes*, plasma estrogen was peaked at 8 months old and significantly declined after 10, 11 and 22 months in females. Spinal bone mineral density (BMD) and micro-architecture by microCT measurement progressively decreased and deteriorated from 8 to 10, 12 and 14 months old, which was more apparent in females than the male counterparts. After 10 months old, *O. latipes* were supplemented with 17α-ethinylestradiol (EE2, a potent estrogen mimic) at 6 and 60 ng/mg fish weight/day for 4 weeks, both reduction in spinal BMD and deterioration in bone micro-architecture were significantly prevented. The estrogenic effect of EE2 in *O. latipes* was confirmed by significant up-regulation of four key estrogen responsive genes in the liver. In general, bone histomorphometric analyses indicated significantly lowered osteoblasts and osteoclasts numbers and surfaces on vertebrae of EE2-fed medaka.

**Significance:**

We demonstrate osteoporosis development associated with natural drop in estrogen level during aging in female medaka, which could be attenuated by estrogen treatment. This small size fish is a unique alternative non-mammalian vertebrate model for studying estrogen-related molecular regulation in postmenopausal skeletal disorders *in vivo* without ovariectomy.

## Introduction

Osteoporosis is a systemic skeletal disease characterized by low bone mass and architectural deterioration with a consequential increase in bone fragility, decrease in biomechanical properties and susceptibility to fractures [1], [2]. Postmenopausal osteoporosis (PMO) commonly occurs in women approximately 10 years after the onset of menopause. In human, depletion of estrogen has been shown as a key factor in pathogenesis of PMO [3], [4], [5], [6]. Supplement with estrogen in postmenopausal women can prevent or attenuate bone loss in postmenopausal women [7].

**Figure 1:**
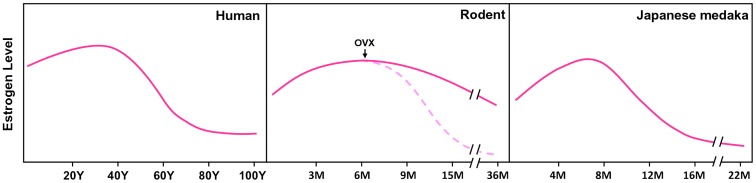
A comparison of estrogen (E2) profiles among aging women, female rodent and female *Oryzias latipes*. Human age is presented in years, and that for both rodent and *Oryzias latipes* is presented in months. Dashed line in rodent panel indicates estrogen level after overiectomy (OVX). Sources: E2 in women by Riggs et al. [34]; E2 in female rodents by Steger and Peluso [8]; E2 in female *Oryzias latipes* by Gopalakrishnan et al. [11] and present study.

Rodent models have long been used for studying PMO for R & D of anti-osteoporosis drugs. However, female rodents have no menopausal drop in estrogen level during aging (Fig. 1) [8]. Therefore, ovariectomy (OVX) has to be conducted in rodents to mimic a natural estrogen decline after menopause in women (Fig. 1) [9], [10]. The imbalanced body homeostasis associated with a sudden loss of estrogen in the artificial OVX animals, which is different from a natural drop of estrogen in postmenopausal women, may influence the precise interpretation of *in vivo* data. Therefore, an alternative vertebrate model with a natural decline of estrogen in females during aging and with similar bone metabolism and therapeutic response as that of the humans would be desirable for R & D for PMO (Fig. 1).

Recently, we reported a natural drop of estrogen level in the small sized laboratory fish, Japanese medaka *Oryzias latipes,* in females during aging, which is akin to “menopause” in women [11]. In addition, *O. latipes* has been well studied for bone cell biology, which is similar to that of the humans [12], [13], [14]. Osteoclasts (OCs) in *O. latipes* are multinucleated, bearing a mammal OC like ruffled border [15], [16]. Our preliminary study of *O. latipes* bone specimen also demonstrated that mammal like osteoblasts (OBs) in cubic or lining forms located on osteoid surfaces as indicated by Trichrome staining. Furthermore, high genomic homology of twist, core binding factor-a1 (*cbfa1*) and osteoprotegerin (*opg*) of OBs were found between *O. latipes* and other vertebrates [17], [18], [19]. Moreover, abnormal hunchback profile is a hallmark of aging in *O. latipes* [20]. The above evidence strongly supported that *O. latipes* could be developed toward an alternative vertebrate model for osteoporosis research, *e.g.* PMO.

Thus far, there is a lack of information on bone metabolism and osteoporosis development in aging *O. latipes*. Our earlier studies have established the *O. latipes* an alternative vertebrate model for sex and aging research [11], [21], [22]. It is hypothesized that estrogen associated disorders in bone metabolism might be predicted and prevented by estrogen supplement in aging *O. latipes*, which could be corresponding to postmenopausal osteoporosis in women. In this study, we first investigated age-related changes in spinal bone mineral density (BMD), bone micro-architecture and plasma estrogen levels in both sexes of *O. latipes*, with an attempt to corroborate plasma estrogen levels and spinal BMD *in vivo*. Subsequently, synthetic 17α-ethinylestradiol (a potent estrogen mimic) was administered as estrogen supplement to study the changes of BMD, bone micro-architecture and histomorphology induced by estrogen in both sexes of 10-month old *O. latipes* (shortly after estrogen decline in the females).

## Materials and Methods

### Ethics Statement

European Union directive 86/609/EEC are strictly adhered throughout the study. Ethics committee specifically approved this study and all animal handling procedures mentioned in this study were accepted by the Animal Ethics Committee, City University of Hong Kong.

### Japanese Medaka Culture

The orange-red line medaka *Oryzias latipes* was originated from the Molecular Aquatic Toxicology Laboratory, Duke University, USA. Fish were transferred to the City University of Hong Kong in 2008. Briefly, 15 pairs of male and female medaka of the same age group were housed in aquaria of dimensions: 39.5L×23.5W×27.5H (cm) and kept under static conditions at 26±1°C, 7.2±0.2 ppm O_2_ under 14 h:10 h light:dark cycle. Fifty percent of tank water was replaced daily with dechlorinated tap water. Fish were fed twice daily with estrogen free diet Otohime β1 (Nisshin Co, Japan), and supplemented with hatched brine shrimp *Artemia nauplii* (Lucky Brand, O.S.I. USA) for 3 days per week. Under the stated husbandry conditions, the fish reach sexual maturity at three months-post-hatch and lay eggs daily. Japanese medaka at different ages: 4 months (young), 8 months (mature), 10–14 months (senior) and 22 months (very old) were sampled for measurement of plasma estrogen by ELISA analysis and bone mass and micro-architecture by microCT analysis.

### EE2 Exposure Experiment: Food Preparation and Administration

17α-ethinylestradiol [EE2] (Cat. No. E4876, Sigma-Aldrich Pty. Ltd), a potent estrogen agonist [23] and more stable in water as compared to the synthetic 17β-estradiol (E2) [24], was used as an estrogen supplement in this study. Two EE2 stock solutions were prepared by dissolving 2.592 and 25.92 mg of EE2 each in 10 mL of absolute ethanol. Dissolved EE2 was mixed with 4.32 g fish feed (Otohime β1) and ethanol was evaporated by nitrogen. The EE2 infused feed was homogenized in distilled water, making two EE2 stock concentrations at 0.012 and 0.12 mg EE2/mL water. The EE2 solutions were aliquot in tubes, stored at −20°C. Control feed was prepared by the same way, except with the omission of EE2.

For the EE2 experiment, fourteen pairs of 10 months old male and female fish (showing a natural drop of estrogen) were used. Each pair of fish was kept in a glass tank having dimensions of 15 cm^3^. Fish was fed daily with EE2 diet or control diet for 4 weeks. EE2 diet was prepared by spiking a 100 µL of high or low EE2 stock solution into 2 mg of fish feed (ca. 1% of body weight). This was equivalent to 6 and 60 ng EE2/mg fish wt./day for low and high dose EE2 treatments, respectively. To avoid uneven consumption of EE2/solvent diet, a glass divider was gently inserted at each tank to separate the fish pair prior to feeding. Upon completion of feed, the glass plate divider was removed. During the experiment, fish were also fed approximately 4 mg of Otohime β1 once daily for growth purpose.

### Fish Sampling

After 4 weeks of EE2 feeding, all fish were anaesthetized by immersing in ice-cold aquarium water for 30 sec. Immediately after anaesthesia, blood sample was collected from individual fish by cutting the tail between 28^th^ and 29^th^ vertebra (i.e. at caudal peduncle). Blood samples were stored at −80°C for estrogen analysis. Fish liver was immediately isolated, snap-frozen in liquid nitrogen and stored at −80°C for qRT-PCR. The remaining fish body was fixed in 4% paraformaldehyde in phosphate buffered saline (PBS) overnight at 4°C, then transferred to 70% ethanol for subsequent microCT scanning and quantitative histomorphometry (n = 6 per sex for each treatment).

### Plasma Estrogen Assay

To extract the plasma estrogen, 5 mL diethyl ether was added to the blood sample, then vortexed and centrifuged at 3000 rpm for 10 min at room temperature. After centrifugation, the organic phase was retrieved using a glass pipette. The extraction procedure was repeated 3 more times for each sample. The level of plasma estrogen was measured using commercially available ELISA Kits (Cat#582251, Cayman Chemical Pty. Ltd.) according to the manufacturer’s instructions. Note that this procedure only detects endogenous plasma estrogen, but not the exogenously added EE2.

### Estrogen Responsive Marker Genes Expression

To confirm the estrogenic effect of EE2 treatment in fish, hepatic expression of estrogen responsive genes including choriogeninH (*ChgH*), estrogen receptor alpha (*ERα*) and vitellogenins (*Vtg1* and *Vtg2*) were measured by qRT-PCR. Isolated liver was homogenized by sterilized micropestle (n = 6 per sex for each treatment). Total RNAs were extracted by RNeasy mini Kit (Qiagen, Germany) and cDNA templates were generated using TaKaRa Primescript™ RT (TaKaRa, China). Briefly, the assay was performed using Master Mix (2x) Universal (KAPA, USA) in ABI 7500 fast system. The primer sets used for qPCR are given in Table 1. The relative mRNA expression level was calculated by the classical 2^−ΔΔ^CT method using 18 s rRNA as an endogenous control to normalize the data.

**Table 1:** Gene specific primers used for quantification by qPCR.

Primers	Sequence	Accession number
*ChgHF*	TACTTTCCCGTCACTTATTGC	D89609
*ChgHR*	TTCCACGACCAGAGTTTCAAC	D89609
*ERαF*	GAGGAGGAGGAGGAGGAGGAG	D28954
*ERαR*	GTGTACGGTCGGCTCAACTTC	D28954
*Vtg1F*	AGTGCTCGTCGTTCAATGC	NM001104677
*Vtg1R*	AGTCGCTGCTTCTGCTTCTA	NM001104677
*Vtg2F*	CACCTGACTACTCCTCTGTTG	NM001104840
*Vtg2R*	GTAATGGAATGCTCTGCTGAAG	NM001104840

### Bone Mass and Micro-architecture Quantified by microCT Analysis

**Figure 2:**
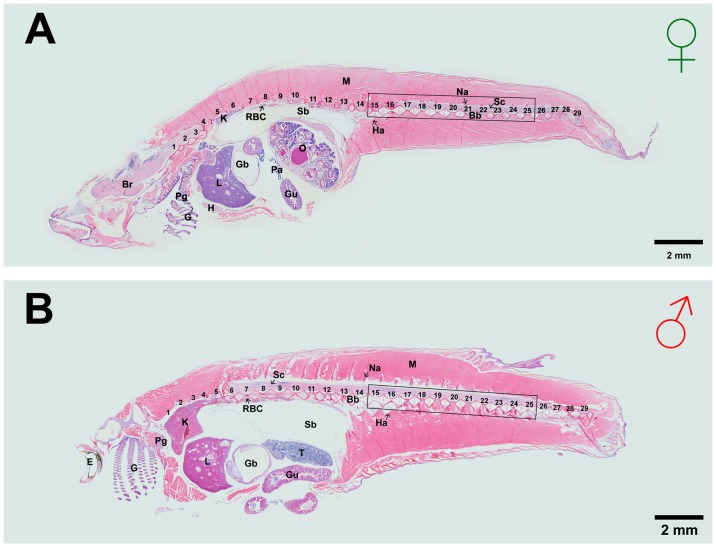
Haematoxylin and Eosin (H and E) staining of sagittal sections from whole adult Japanese medaka *Oryzias latipes* in (A) female and (B) male at 13 months old. Fish anterior is to the left. Spinal vertebrae are numbered. The rectangular box showing the vertebrae 15^th^–25^th^ was used for both microCT scanning and histomorphometry. Letters on the sections indicate Br, Brain; Bb, Backbone; E, Eye; G, Gill; Gb, Gall bladder; Gu, Gut; H, Heart; Ha, Hemal arch; K, Kidney; L, Liver; M, Muscle; Na, Neural arch; O, Ovary; Pa, Pancreas; Pg, Pharyngeal gill; RBC, Red Blood Cells, Sb, Swim bladder; Sc, Spinal cord; T, Testis.

The bone mass and micro-architecture was measured by microCT (µCT40, SCANCO MEDICAL, Switzerland). Briefly, the whole body of medaka was fixed in a plastic tube (SCANCO MEDICAL, Switzerland) of 5 mm diameter and scanned for scout view. The 15^th^ to 25^th^ vertebrae region (Fig. 2) were selected for scanning with a voxel size of 6 µm. Totally, 900 continuous slices (each slice with a thickness of 6 µm) were selected and all the mineralized bone from each selected slice was segmented for three-dimensional reconstruction of micro-architecture and analysis of volumetric bone mineral density (BMD) (Sigma = 1.2, Supports = 2 and Threshold = 160).

### Bone Histology

Whole medaka fish were fixed and processed according to the protocol described by Kong et al. [25], which allows bone histology without decalcification. Briefly, adult medaka were anaesthetized and dissected, hard substances including skull, otoliths and fins removed, and fixed in GPHS [0.05% glutaraldehyde, 2% paraformaldehyde, 80% histochoice MB fixative (Cat# 64115-04, Electron Microscopy Sciences, USA), 1% sucrose and 1% CaCl_2_] fixative for 48 h. Fixed fish were dehydrated in series of methanol and chloroform and embedded in paraffin. Serial sections of a single fish were cut (7 µm) using rotary microtome (Leica RM2125RT, Germany). Sections were mounted onto SuperFrost® Plus slides (Menzel-Gläser, Germany) and dried at 33°C overnight. Whole fish sections were observed under compound microscope, sections showing vertebral columns were chosen for staining. Solvent control and EE2 treated sections were collected and number coded for quantitative histochemical staining of OBs and OCs.

### Identification and Localization of OBs and OCs by Goldner’s Trichrome and TRAP Staining, Respectively

Osteoblasts (bone forming cells) were detected and localized by Alkaline Phosphatase (ALPase) activity using the Goldner’s Trichrome modified staining protocol. Briefly, deparaffinized medaka tissue sections were placed in Weigert’s haematoxylin for nuclei staining (15 min). The tissue sections were put in Ponceau/acid fuchsin/azaphloxine for 5 minutes, followed by differentiation with 0.5% acid alcohol for 20 sec. The stained sections were subject to phosphomolybdic acid and light green treatment for 5 and 20 minutes, respectively, prior to dehydration in series of ethanol and xylene.

Osteoclasts (bone resorbing cells) were localized by Tartrate Resistant Acid Phosphatase (TRAP) enzyme activity using acid phosphatase, leucocyte (387A, Sigma-Aldrich) staining protocol according to the manufacturer’s instructions (Sigma-Aldrich). The stained sections were briefly air dried and mounted in permount (SP15-500, Fisher scientific) for further analyses.

All stained sections were examined under light microscopy [40x objective magnification] (Axioplan 2 Imaging, Germany) and images were captured using a color viewII camera (Soft Imaging Solutions, GmbH, Germany) for identification of OBs and OCs.

### Quantification of Number and Surface of OBs and OCs by Bone Histomorphometric Analyses

Four bone histomorphometric parameters (i) osteoblasts number/mm of bone perimeter (Ob.N/B.Pm), (ii) osteoclasts number/mm of bone perimeter (Oc.N/B.Pm), (iii) osteoblasts surface/bone surface (Ob.S/BS) and (iv) osteoclasts surface/bone surface (Oc.S/BS) were quantified by Bioquant Osteo v13.1.60 image analysis software (BIOQUANT Image Analysis Corporation, USA). The above parameters were measured and expressed according to the standardized nomenclature for bone histomorphometry [26].

### Statistical Analyses

The data of estrogen levels was analyzed by Kruskal-Wallis test using the Graphpad prism 6.01 v package. The data for estrogen responsive gene expressions, microCT and bone histomorphometric parameters in aging/EE2 treatment studies were analyzed among groups by one-way ANOVA and followed by Tukey’s post-hoc test to determine the sex and treatment effect using SPSS package (SPSS Inc. 2008) at P<0.05 significance.

## Results

### Declined Estrogen Level in Aging *O. latipes*

**Figure 3:**
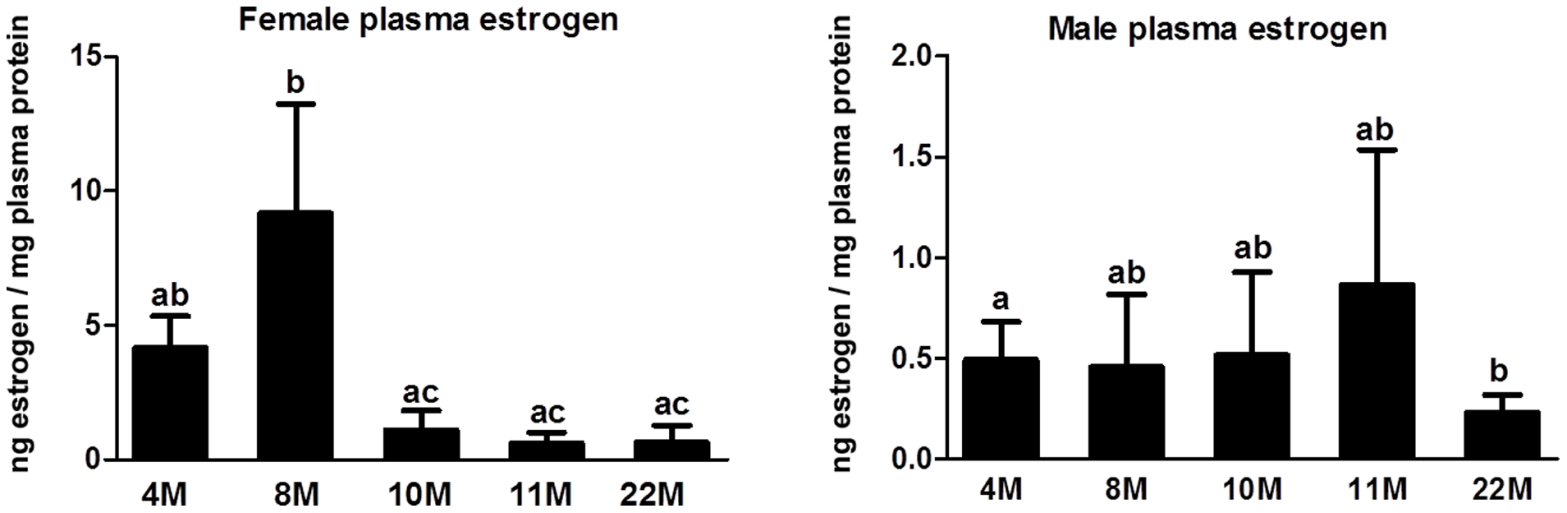
Plasma estrogen levels in aging female (left) and male (right) Japanese medaka O*ryzias latipes*. Bars labeled by the same letter (a, b, c) on the graph are not significantly different from each other (p<0.05) by one-way ANOVA followed by Tukey’s post hoc test.

Plasma estrogen levels in 4, 8, 10, 11, and 22 months old *O. latipes* were analyzed for both sexes. In females, the estrogen level was peaked at 8 months old, declined rapidly at 10–11 months old (P<0.05 and P<0.001, respectively) and remained low at 22 months old (P<0.0001) (Fig. 3A). In males, the estrogen level was lower than the females and declined gradually from 4 to 22 months old (Fig. 3B).

### Decreased Bone Mass in Aging *O. latipes*

**Figure 4:**
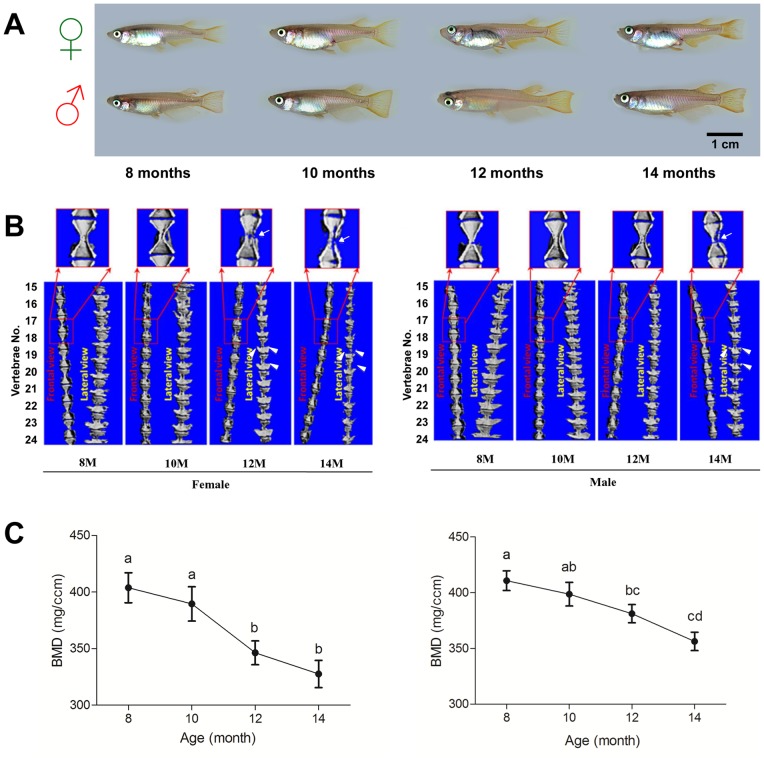
Representative appearance of female (top) and male (bottom) Japanese medaka *Oryzias latipes* at 8, 10, 12 and 14 months old (Fig. A). (B) Representative micro-architecture of vertebrae bodies at 8, 10, 12 and 14 months in females (left) and males (right) by 3-dimensional microCT reconstruction. (C) Age-related changes in bone mineral density (BMD) from 8 to 14 months in females (left) and males (right) by microCT analysis. White arrows and arrowheads indicate micro-cracks and thinner arches found in 10–14 months old fish. BMD values labeled by the same letter (a, b, c, d) on the graph are not significantly different from each other (p<0.05).

The BMD and micro-architecture of 15^th^∼25^th^ vertebrae bodies were measured by microCT for both genders at four different age groups (8, 10, 12 and 14 months old) (Fig. 4A). As revealed by three dimensional reconstruction images using microCT, the micro-architecture of the vertebrae bodies deteriorated, including the formation of micro-cracks (white arrows), thinner arches (hemal and neural) (white arrowheads) and progressively curved vertebral column (Fig. 4B), which was obvious in females at 12 months old onwards and in males at 14 months. The BMD in females declined from 8 to 10 months old and reached significantly lower level at 12 and 14 months (P<0.05) (Fig. 4C, left). In males, the BMD declined gradually with age and towards significantly lower level at 12 months onwards compared to 8 and 10 months (Fig. 4C, right). Age-associated decline of BMD was faster in females than males after 10 months old (Fig. 4C).

### Estrogenic Effects in *O. latipes* after EE2 Treatment

**Figure 5:**
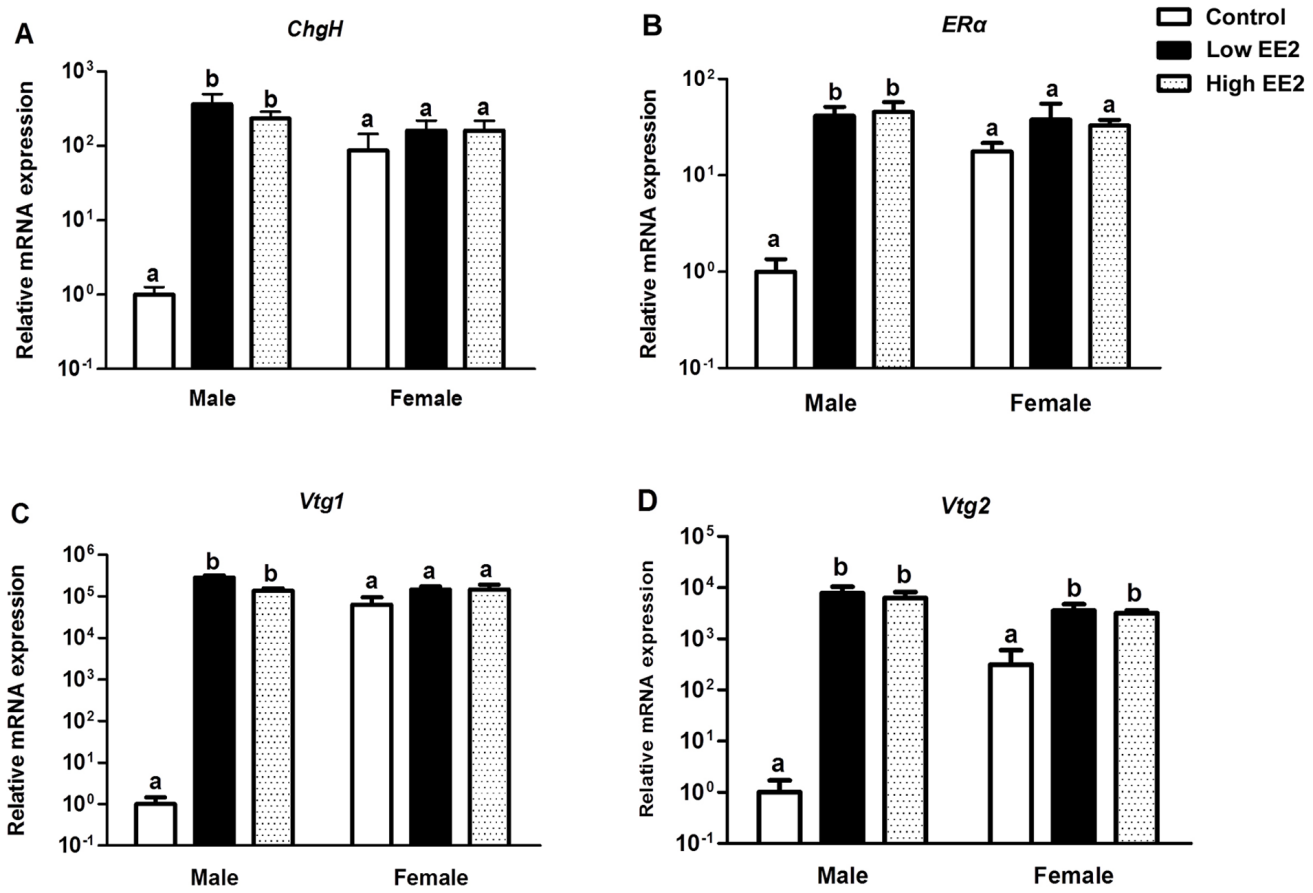
Hepatic expression of estrogen responsive genes in male and female *Oryzias latipes* treated with low and high EE2 for 4 weeks. (A) Choriogenin H (*ChgH*), (B) Estrogen Receptor α (*ER*α), (C) Vitellogenin1 (*Vtg1*), (D) Vitellogenin2 (*Vtg2*). Bars labeled by the same letter on the graphs are not significantly different from each other (p<0.05).

The estrogenic effect of EE2 was confirmed by significant up-regulation of estrogen responsive marker genes in liver of EE2 treated fish (Fig. 5 A to D). In males, *Vtg1* was the most sensitive marker (ca. 1.4–2.9×10^5^ folds), followed by *Vtg2* (ca. 7×10^3^ folds), *ChgH* (ca. 400 folds) and *ERα* (ca. 40 folds) for both EE2 treatments. In females, probably due to high endogenous expressions of all four estrogen responsive genes, only *Vtg2* expression was significantly up-regulated upon EE2 treatment (ca. 10 folds) (Fig. 5D).

**Figure 6:**
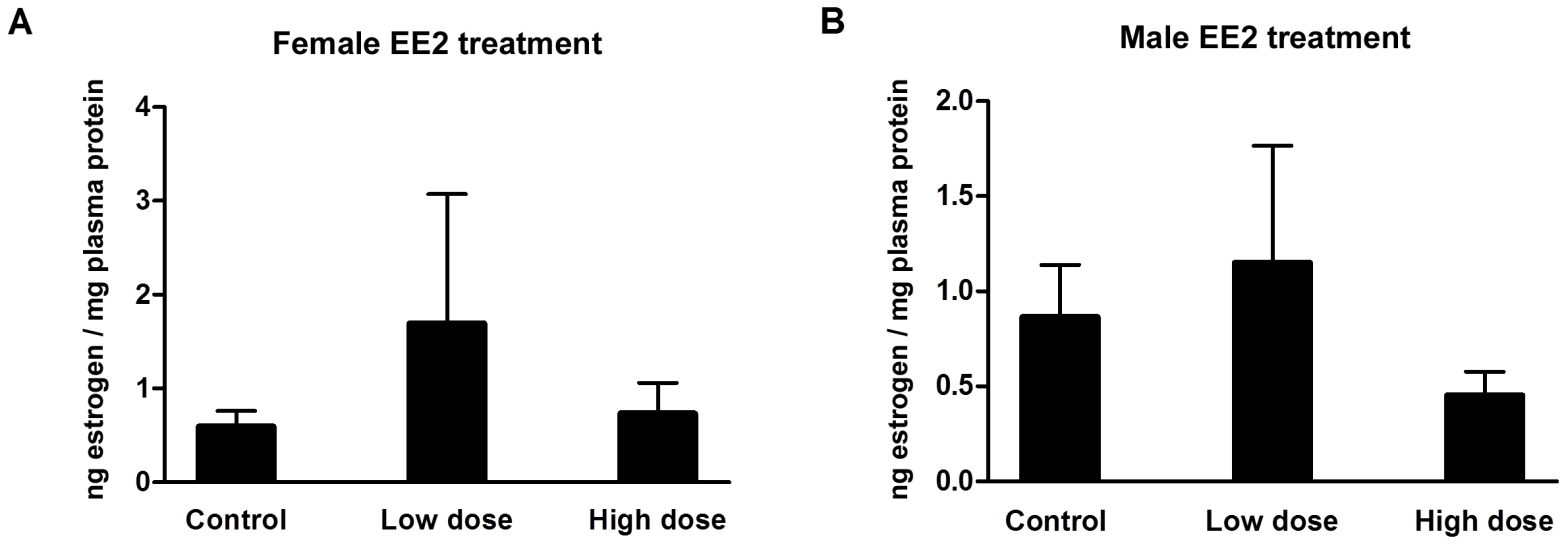
Plasma estrogen in 10 months old female (A) and male (B) Japanese medaka *Oryzias latipes* treated with low and high EE2 for 4 weeks. No significance at P<0.05.

We also tested the effect of EE2 addition on the endogenous plasma estrogen levels. While these levels varied among fish within each EE2 treatment group, no significant change observed (Fig. 6 A, B).

### Improved Micro-architecture and Prevented BMD Decline after EE2 Treatment

**Figure 7:**
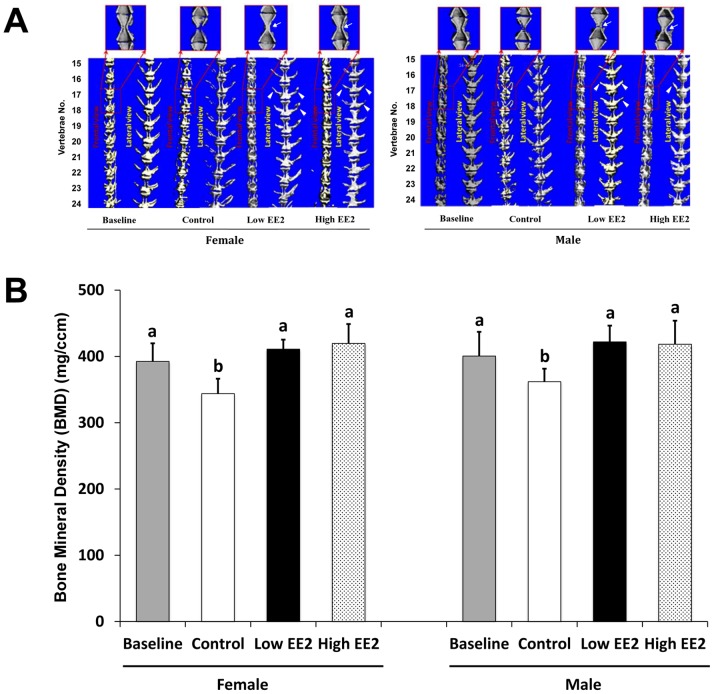
Changes in micro-architecture (A) and bone mineral density (BMD) (B) of vertebrae bodies from 10 months old (baseline) to 11 months old female and male Japanese medaka *Oryzias latipes* after low and high EE2 treatment for 4 weeks. White arrows and arrowheads indicate improved micro-architecture and arches in EE2 treated fish. Bars labeled by the same letter (a, b) on the graph are not significantly different from each other (P<0.05).

A better organized micro-architecture, including less micro-cracks (white arrows), thicker arches (hemal and neural) (white arrowheads) and also no abnormal vertebral column curvature, was found in EE2 treated fish for both genders (Fig. 7A).

EE2 treatment could prevent bone loss in aging *O. latipes*. Under normal condition, between the 10 months old “Baseline” and the 11 months old “Control”, the spinal BMD declined from an average of 392 to 344 mg/ccm (12.2%) and 401 to 362 mg/ccm (9.7%) in females and males, respectively (n = 6 per group) (Fig. 7B). Upon EE2 treatment for 4 weeks, BMD in both low and high EE2 treated females was significantly higher than the control females and showed no difference to the “Baseline” (P<0.05, n = 6 per group) (Fig. 7B). Similar trend was found in males (P<0.05) (Fig. 7B).

### Changes in Bone Turnover (Bone Formation and Resorption) after EE2 Treatment in *O. latipes*

**Figure 8:**
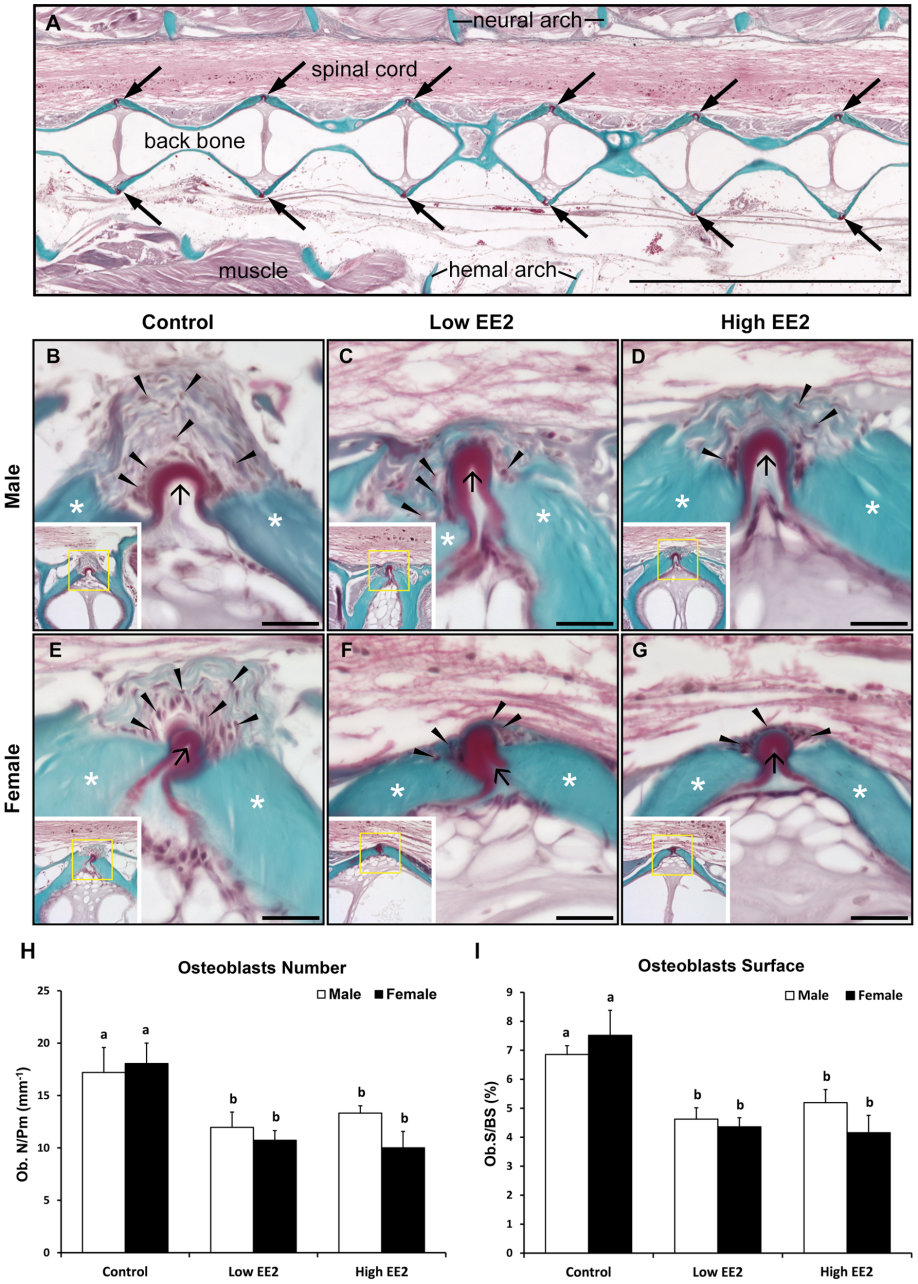
Representative histology pictures of Goldner’s Trichrome staining for detection of osteoblasts (black arrowheads), osteoid (black arrows) and mineralized bone (asterisks) in both sexes of Japanese medaka *O. latipes* after 4 weeks of EE2 treatment (Control = no EE2 fed; Low = 6 ng EE2/mg body wt./day and High = 60 ng EE2/mg body wt./day) (Fig. A–G). (A) The location of the corresponding cells on the serial tissue sections is indicated by arrows (scale bar 1000 µm). (B) Male untreated/Control, (C) Male with low EE2 treatment, (D) Male with high EE2 treatment, (E) Female untreated/Control, (F) Female with low EE2 treatment and (G) Female with high EE2 treatment. Scale bars 20 µm. Figure 8 H and I. Bone histomorphometric parameters in both sexes of *O. latipes* upon EE2 treatment (H) Osteoblasts Number/Bone Perimeter (mm)(Ob.N/B. Pm), (I) Osteoblasts Surface/Bone surface (Ob.S/BS). Bars labeled by the same letter on the graphs are not significantly different from each other (At significant level p<0.05).

Bone histomorphometry results by Goldner’s Trichrome staining indicate changes in OBs numbers (arrowheads) on the bone surfaces around the osteoid (black arrows) and OBs surface (asterisks on green surface indicate mineralized bone) were quantified between 15^th^–25^th^ vertebrae in all EE2 experimental male and female *O. latipes* (Fig. 8 B–G). In males, low and high EE2 treatments induced significantly lower OBs numbers and surfaces as compared to control (Fig. 8 H, I). Similarly, in females, OBs numbers and surfaces were significantly lower in both low and high EE2 treatments compared to controls (Fig. 8 H, I). Further, no sex differences for OBs numbers and surfaces were observed in the controls and EE2 treatment groups (Fig. 8 H, I).

**Figure 9:**
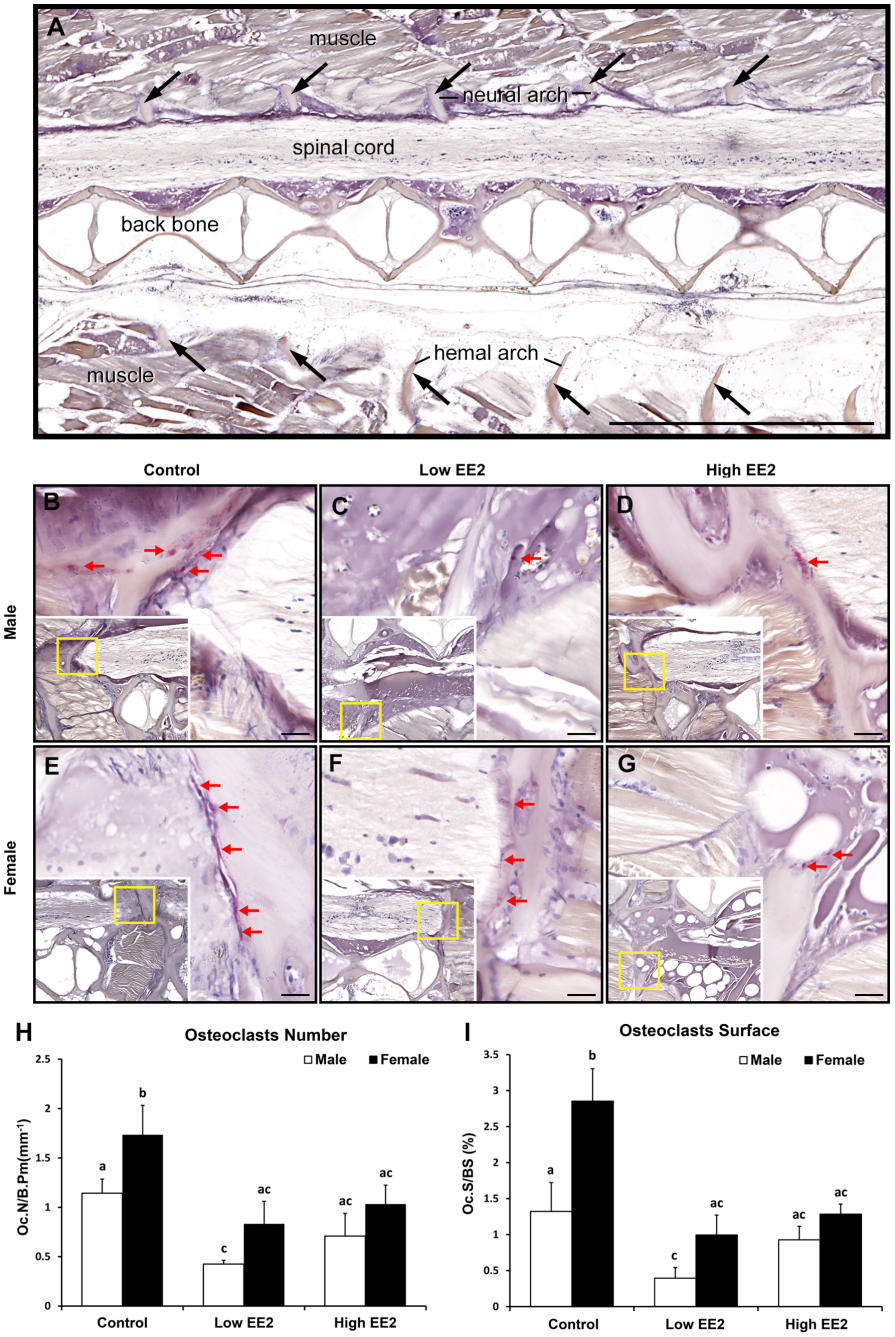
Representative histology pictures of TRAP staining for detection of osteoclasts in both sexes of Japanese medaka *O. latipes* after 4 weeks of EE2 treatment (Control = no EE2 fed; Low = 6 ng EE2/mg body wt./day; High = 60 ng EE2/mg body wt./day) (Fig. A–G). (A) The location of the corresponding cells on the serial tissue sections is indicated by arrows (scale bar 1000 µm). (B) Male untreated/Control, (C) Male with low EE2 treatment, (D) Male with high EE2 treatment, (E) Female untreated/Control, (F) Female with low EE2 treatment and (G) Female with high EE2 treatment. Red arrows on histology sections indicate TRAP signals. Scale bars 20 µm. Figure 9 H and I. Bone histomorphometric parameters on vertebrae in both sexes of *O. latipes* upon EE2 treatment (H) Osteoclasts Number/Bone Perimeter (mm) (Oc.N/B. Pm) and (I) Osteoclasts Surface/Bone Surface (Oc.S/BS). Bars labeled by the same letter on the graphs are not significantly different from each other (At significant level p<0.05).

Bone histomorphometry results by TRAP staining showed that EE2 treatment lowered OCs numbers (red arrows) in both sexes of *O. latipes* as compared to their respective controls (Fig. 9 B–G). The OCs numbers were quantified on hemal and neural arches of the vertebrae between 15^th^–25^th^ vertebrae. In males, the OCs numbers and surfaces were significantly lower only in the low EE2 treatment as compared to the control (Fig. 9 H, I). Whereas, in females, OCs numbers and surfaces were significantly lower in both low and high EE2 treatment as compared to the control (Fig. 9 H, I). Sex differences in OCs numbers and surfaces were only observed in the controls, where female >male (Fig. 9 H, I).

Overall, a concomitantly lowered bone formation and resorption indicates reduction in bone turnover in *O. latipes* after 4 weeks of EE2 treatment.

## Discussion

In the current study, for the first time we demonstrate osteoporosis development associated with natural drop in estrogen level during aging in female medaka, which could be attenuated by estrogen treatment.

### Osteoporosis Development Associated with Natural Drop in Estrogen Level in Medaka

Here we report striking similarities between female *O. latipes* and women in age-dependent natural drop of estrogen [11], and subsequent deterioration of bone metabolism including decreased BMD, poor organized bone micro-architecture and high bone turnover. Such changes were also observed in male medaka, but gradually occurred.

Using the medaka-human age conversion model developed by our team [11], females at 8- and 10- months old, showing a drastic decline in plasma estrogen level after the peak level at 8-month, could be corresponding to women at 46- and 55- years old (postmenopausal age) [3]. Further study should be conducted using a wide spectrum of teleosts to ascertain whether estrogen decline associated osteoporosis is a general phenomenon in bony fish during aging. Perhaps this could shed light on such conserved fundamental mechanism during evolution from lower vertebrates to human.

We also observed gender difference in bone mass and micro-architecture deterioration in medaka, which occurred later in the males (Fig. 4B, see arrows in 14 months panel) as compared to the females (Fig. 4B, see arrows in 12 and 14 months panel). A two months difference in medaka age (12 months vs 14 months) is equivalent to a 10 years difference of human age (60 vs 70 years old) [11]. In another study, a significant decrease in estrogen level was observed after 11 months in males [11]. Therefore, it is clear that the osteoporosis prevalence in medaka is delayed in males compared to females. The prevalence of osteoporosis in male medaka also corresponded with a significant decline in estrogen level in males after 12 months of age [11]. The present study further illustrates a gender difference (female>male) in OCs numbers and surfaces (Fig. 9G, H), not OBs (Fig. 8G, H), which could explain a higher bone mass in males as compared to the female.

Our results are in agreement with earlier reports that OCs were only found on hemal and neural arches, but not on the main vertebrae in *O. latipes,* unlike those reported findings in other mammalian studies [14], [27].

### Estrogen Treatment Attenuated Osteoporosis Development in Medaka

The numerical values of plasma estrogen in females were higher than controls after EE2 treatment, but no statistical significance was observed due to large variation. This response is not surprising due to the very complex feedback mechanisms in regulation of estrogen level *in vivo* [28]. Nevertheless, the estrogenic effect due to supplementation of exogenous EE2 in *O. latipes* was confirmed by up-regulation of four key estrogen responsive genes (*ERα, Vtg1, Vtg2* and *ChgH*) in the liver.

EE2 treatment could attenuate decreased BMD and deteriorated bone micro-architecture observed in the spine during aging (between the 10 months old “Baseline” vs the 11 months old “Control”) in both sexes. This could be explained by multiple levels of evidence, at the molecular (estrogen responsive gene expressions), cellular (quantitative bone histomorphometry) and organ level (BMD and micro-architecture by microCT scanning) obtained in the current study. Mechanistically, quantitative histomorphometry analyses clearly evidenced a concomitant reduction in OBs and OCs numbers and surfaces on vertebrae of EE2 treated *O. latipes,* suggesting EE2-induced slowdown in bone turnover. The above findings were consistent with observations in postmenopausal women with osteoporosis after estrogen replacement therapy (ERT) [7], [29], [30], [31]. Overall, a concomitantly lowered bone formation and resorption in *O. latipes* indicates a lowered ‘Bone turnover’ after 4 weeks of EE2 treatment.

This is the first report that EE2 treatment could be effective in inducing improvement on estrogen declined associated bone disorders in teleost. However, it is still prudent to understand the prolonged treatment effect on bone metabolism in bony fish.

### Estrogen Mediated Regulation of Bone Metabolism based on *in vitro* and *in vivo* Findings in Mammals

**Figure 10:**
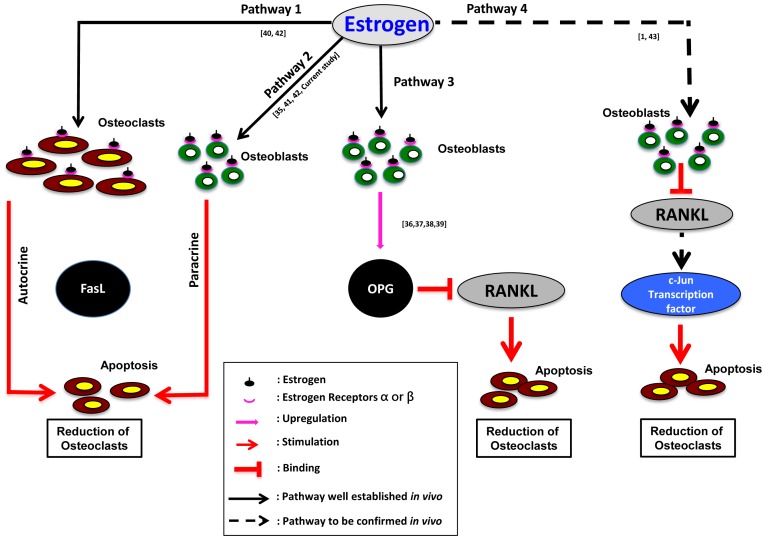
Schematic diagram showing estrogen mediated regulation of bone metabolism based on *in vitro* and *in vivo* findings in mammals. FasL: Fatty acid synthatase Ligand, RANKL: Receptor Activator of Nuclear factor Kappa-B Ligand, OPG: Osteoprotegerin. Dark arrows indicate up-regulation; Red arrows indicate down-regulation/apoptosis of osteoclasts. Please refer to the details in

Several molecular mechanisms have been postulated on estrogen-mediated modulation of bone metabolism, based on *in vivo* and *in* *vitro* evidence using mammals, which are summarized in Figure 10 [35], [36], [37], [38], [39], [40], [41], [42], [43]. These mechanisms may be conserved in vertebrates from fish to mammals, which warrant further investigations.

### The Translational Significance of the Findings

This work provides a unique, non-mammalian vertebrate model with natural estrogen decline for biomedical research on postmenopausal osteoporosis *in vivo* without the need of invasive overiectomy. Further, findings of the present study support the development of small sized *O. latipes* as an alternative, cost-effective vertebrate model for not only studying disorders in bone metabolism, but also R & D for drugs screening.

On the other hand, *O. latipes* has been a well-established *in vivo* laboratory model for toxicological research [32]. From the perspectives of environmental toxicology, the understandings of bone biology in *O. latipes* will contribute greatly to unravel the mechanisms underlying the high incidences of fish skeletal deformation in polluted waters [33]. We anticipate the current findings on estrogen and bone metabolism in fish will also facilitate exploring future research on evolutionary biology in vertebrates.

## Supporting Information

Text S1 (PDF)
