# Supplementary material for: Japanese Medaka: A Non-Mammalian Vertebrate Model for Studying Sex and Age-Related Bone Metabolism In Vivo
Source: PLoS One. 2014 Feb 11;9(2):e88165. doi: 10.1371/journal.pone.0088165 (PMC3921145; doi:10.1371/journal.pone.0088165)
Supplement: Text S1 — (PDF) [file pone.0088165.s001.pdf]

The specific mechanisms involved in estrogen modulation of OBs and OCs, however, remains unclear in teleosts. Yet, similar modulation was also observed in rats treated with 17β-estradiol [35] as observed in the present study. In addition, bone morphogenetic protein-2 (BMP-2) gene expression could be elevated by estrogen via estrogen receptors (ERs) by binding to the estrogen responsive elements (ERE) on the BMP-2 promoter in OBs [31]. As for OCs regulation, earlier studies documented that the receptor activator of nuclear factor kappa B ligand (RANKL) is a key regulator of OC differentiation, function and survival, which is controlled via osteoprotegerin (OPG) produced by the OBs [36]. It has been speculated that estrogen suppresses OCs by up-regulating OPG that blocks RANKL [36,37,38,39]. Further, estrogen was also shown to up-regulate FasL gene (via ERs on OBs and OCs, through autocrine and paracrine mechanism), a key contributor for OC apoptosis [40, 41, 42]. Besides, estrogen also regulates (suppress) OCs through c-Jun transcription factor by repressing OC precursors differentiation through the inhibition of RANKL [1, 43]. Therefore, the significance of above pathways should be considered in future studies to unravel the molecular action of EE2 on regulating OBs and OCs role in bone turnover in *O. latipes* or could be for any other teleosts.
